# Desmoplastic Small Round Cell Tumor in a Young Adult: A Case Report Highlighting Diagnostic Challenges and Treatment Approaches

**DOI:** 10.7759/cureus.69873

**Published:** 2024-09-21

**Authors:** Nikhil Puri, Sabrina Carpintieri, Agostino Grittani, Nicole Flaherty, Raul Cortinas

**Affiliations:** 1 Surgery, St. George’s University School of Medicine, True Blue, GRD; 2 Surgery, Ross University School of Medicine, Miami, USA; 3 Internal Medicine, St. George’s University School of Medicine, True Blue, GRD; 4 Pediatrics, St. George’s University School of Medicine, True Blue, GRD; 5 Internal Medicine, Jackson Memorial Hospital, Miami, USA

**Keywords:** desmoplastic small round cell tumor, dsrct, multimodal therapy, peritoneal carcinomatosis, soft tissue sarcoma

## Abstract

Desmoplastic small round cell tumor (DSRCT) is a rare and aggressive soft tissue sarcoma that predominantly affects young adults. We present the case of a 25-year-old African American male with a recent diagnosis of advanced DSRCT. The patient initially had a prolonged period of nonspecific abdominal symptoms for over a year before he was diagnosed. The patient presented with abdominal pain, abdominal bloating, ascites, constipation, and back pain. Upon admission, imaging studies revealed extensive peritoneal carcinomatosis, a large pelvic mass, and metastatic spread to the lung and lymph nodes. The diagnosis was confirmed through retroperitoneal lymph node biopsy and immunohistochemistry. The specimen showed a characteristic immunophenotype which was CD56 positive and contained the dot-like desmin staining. The Ki67 proliferation rate was greater than 90% indicating that the tumor was highly aggressive. Treatment was initiated using a multimodal approach, which included intensive chemotherapy. The patient was placed on a regimen alternating the combination of vincristine, doxorubicin, and cyclophosphamide for one week and ifosfamide and etoposide for the next. This case spotlights the challenges in early diagnosis of DSRCT and highlights the importance of increased suspicion in young adults who present with vague abdominal complaints. It also discusses the complexity and challenges of managing this rare aggressive malignancy. This case report also addresses the necessity for advanced research into targeted therapies and optimized strategies in the treatment to help improve the survival rates and quality of life for patients with DSRCT.

## Introduction

Desmoplastic small round cell tumor (DSRCT) is an extremely rare and aggressive soft tissue sarcoma that primarily affects adolescents and young adults [1,2]. Since its initial description by Gerald and Rosai in 1989, the number of reported cases has increased significantly, with current literature suggesting over 1,500 documented cases globally [2,3].

DSRCT typically originates from the serosal surfaces of the abdominal cavity, with the peritoneum being the most common primary site. However, cases have been documented in various locations, including the thoracic cavity, central nervous system, and extremities [4,5]. The disease demonstrates a strong male predominance, with recent epidemiological data showing that males are affected approximately four times more frequently than females. Additionally, there appears to be a higher incidence rate among Black individuals compared to other racial groups [5,6].

Genetically, DSRCT is characterized by a unique chromosomal translocation t(11;22)(p13;q12), resulting in the EWSR1-WT1 gene fusion [7]. This genetic alteration leads to the formation of a chimeric transcription factor that increases the expression of several growth factors and transcriptional activators, contributing to the tumor’s aggressive behavior [5]. The EWS-WT1 fusion product specifically induces the expression of platelet-derived growth factor A (PDGFA), which plays a crucial role in the tumor’s pathogenesis [8]. PDGFA is responsible for collagenous stromal production, inflammatory cell infiltration, and neo-angiogenesis. It also induces proliferation and acts as a chemoattractant to fibroblasts and endothelial cells, explaining the characteristic desmoplastic stroma surrounding nests of tumor cells in DSRCT [8,9].

Clinically, DSRCT often presents with nonspecific symptoms such as abdominal pain, distention, and weight loss. Due to its initially subtle and slowly progressing course, patients are frequently diagnosed at an advanced stage with extensive peritoneal involvement and metastatic spread. The current standard of care involves a multimodal approach, combining aggressive surgical resection, intensive chemotherapy, and radiation therapy.

Despite recent advancements in treatment strategies, the prognosis remains poor. The five-year overall survival rate is estimated to be less than 15%, with most patients succumbing to the disease within three years of diagnosis [10].

## Case presentation

A 25-year-old African American male was referred to the emergency department by his primary care physician. The patient’s medical history was unremarkable, except for a third-degree burn on his abdomen at age four. No family history of cancer or other relevant conditions was reported.

The patient’s symptoms had been developing over time: a one-year history of back pain, a three-month history of abdominal distention, and a two-week history of worsening constipation, increased abdominal pain, exacerbation of back pain, abdominal bloating, and decreased appetite. These symptoms had been managed independently until their recent exacerbation prompted medical attention.

Upon presentation to the emergency department, the patient’s vital signs were measured and documented (Table 1). Simultaneously, a comprehensive panel of laboratory tests was performed (Table 2). The patient was tachycardic with a heart rate of 119 beats/minute, while other measurements including temperature, blood pressure, respiratory rate, and oxygen saturation were within normal limits. His initial lab results had multiple abnormalities, including elevated venous pH, low pCO_2_, hyperkalemia, low carbon dioxide, and low osmolality. Liver function tests showed elevated direct bilirubin (4 mg/dL), and elevated aspartate transaminase and alanine aminotransferase levels (60 U/L and 27 U/L, respectively). Hematology results indicated low hemoglobin, high platelet count (726 × 10^3^/µL), and elevated neutrophils. Notably, the elevated high white and red blood cell counts made this ascitic fluid of an exudative nature. Tumor markers appeared to be within normal ranges.

**Table 1:** Patient vitals upon admission.

Vital signs recorded	Recorded values on admission
Temperature	36.9°C
Blood pressure	114/92 mmHg
Heart rate	119 beats/minute
Respiratory rate	18 breaths/minute
SpO_2_	96%

**Table 2:** Initial laboratory investigation. H indicates a high value outside the reference range; L indicates a low value outside the reference range; ! indicates a critically high/low value; N indicates a normal value. pCO_2_: partial pressure of carbon dioxide; pO_2_: partial pressure of oxygen; HCO_3_: bicarbonate

Lab results	Results	Reference range	Units
Blood gas			
pH, venous	7.43 (H)	7.32–7.42	N/A
pCO_2_, venous	38 (L)	42–52	mmHg
pO_2_, venous	44 (N)	30–50	mmHg
HCO_3_, venous	25 (N)	22–29	mEq/L
Hemoglobin oxygen saturation	79.5 (L) (!)	95–100%	%
General chemistry			
Sodium	135 (L)	135–145	mEq/L
Potassium	6.2 (H)	3.5–5.0	mEq/L
Carbon dioxide	21 (L)	22–29	mEq/L
Osmolality	269 (L)	275–295	mOsm/kg
Direct bilirubin	4 (H) (!)	0.0–0.3	mg/dL
Aspartate aminotransferase	60 (H)	10–40	U/L
Alanine aminotransferase	27 (N)	7–56	U/L
Alkaline phosphatase	24 (L)	44–147	U/L
Lactate dehydrogenase	264 (H)	140–280	U/L
Hematology			
Red blood cell count	4.95 (N)	4.5–5.9	×10^6^/µL
Hemoglobin	13.1 (L)	13.5–18	g/dL
Platelet	726 (H) (!)	140–400	×10^3^/µL
Neutrophil	73.3 (H)	40–60	%
Absolute neutrophil	6.2 (H)	2–6.0	×10^3^/µL
Tumor markers			
Alpha-fetoprotein	2.9 (N)	0–9	ng/mL
Cancer antigen 19-9	1.7 (N)	0–37	U/mL
Carcinoembryonic antigen	1.1 (N)	0–2.5	ng/mL
Ascitic fluid analysis			
Appearance	Clear	Clear, straw colored	N/A
White blood cell	356 (H)(!)	0–5	cells/mm³
Red blood cell	1,702 (H)	<1,000	mm³
Neutrophils	5% (H)	0	%

Physical examination revealed an alert patient in no acute distress. The abdomen was bloated showing signs of ascites, with pain noted in the right upper quadrant during palpation. Two wounds were noted on the abdomen and a lesion was observed on the right greater toe.

**Figure 1:**
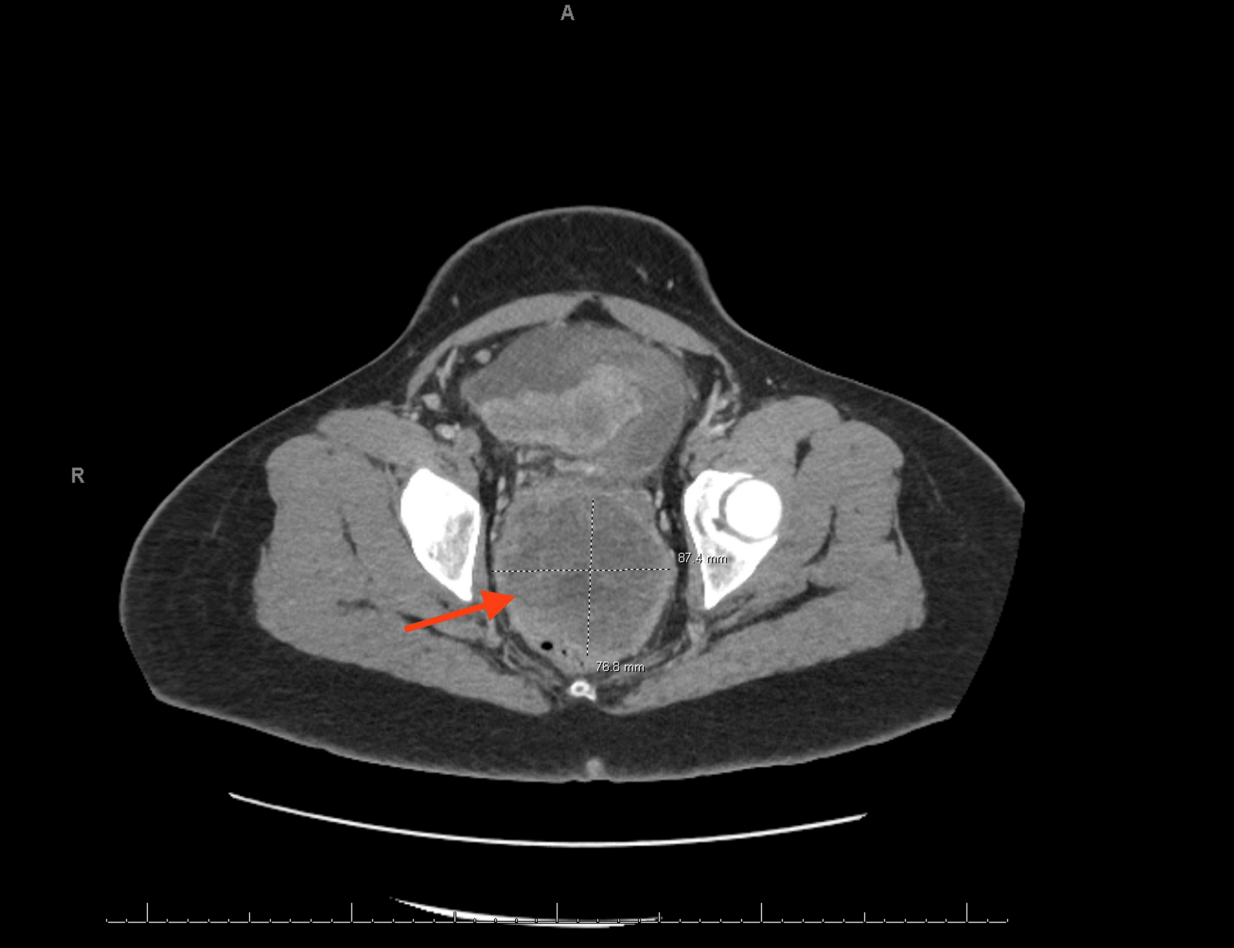
CT of the pelvis with contrast. The image displays the chest CT scan with contrast, and the arrow indicates the pelvic mass.

Imaging studies were conducted, including an abdominal CT scan, MRI of the pelvis, and CT of the chest with contrast. An abdominal CT scan revealed a heavy burden of peritoneal carcinomatosis with a dominant pelvic mass measuring greater than 10 cm, contiguous with the anterior wall of the rectosigmoid. The scan also showed multi-station thoracic and abdominopelvic lymphadenopathy, a moderate volume of abdominal and pelvic ascites, and indeterminate tiny lung base nodules (Figure 1).

**Figure 2:**
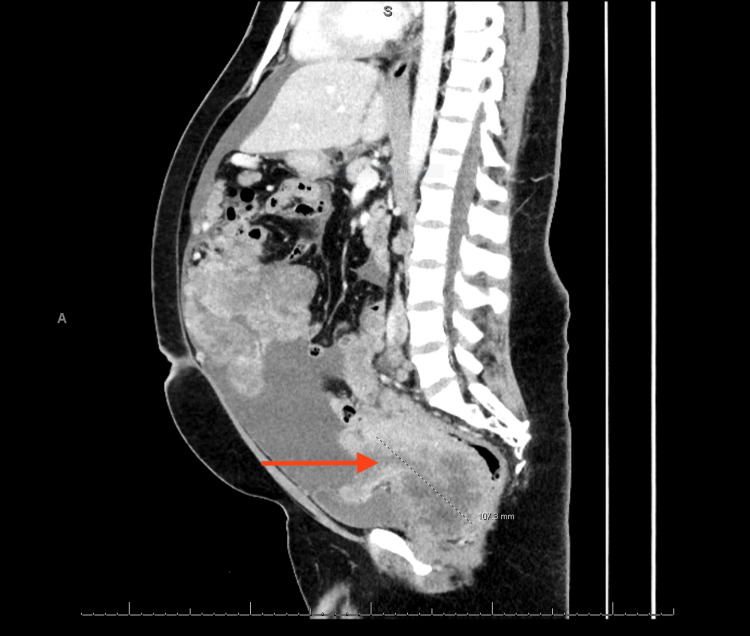
MRI of the abdomen (sagittal cut). The image displays a sagittal abdominal MRI. The red arrow indicates the tumor.

An MRI of the pelvis showed widespread heterogeneously enhancing peritoneal and omental soft tissue masses with central necrosis. The largest lesion in the posterior pelvis measured 14 × 10 × 16 cm, surrounding and narrowing the rectum lumen (Figure 2).

**Figure 3:**
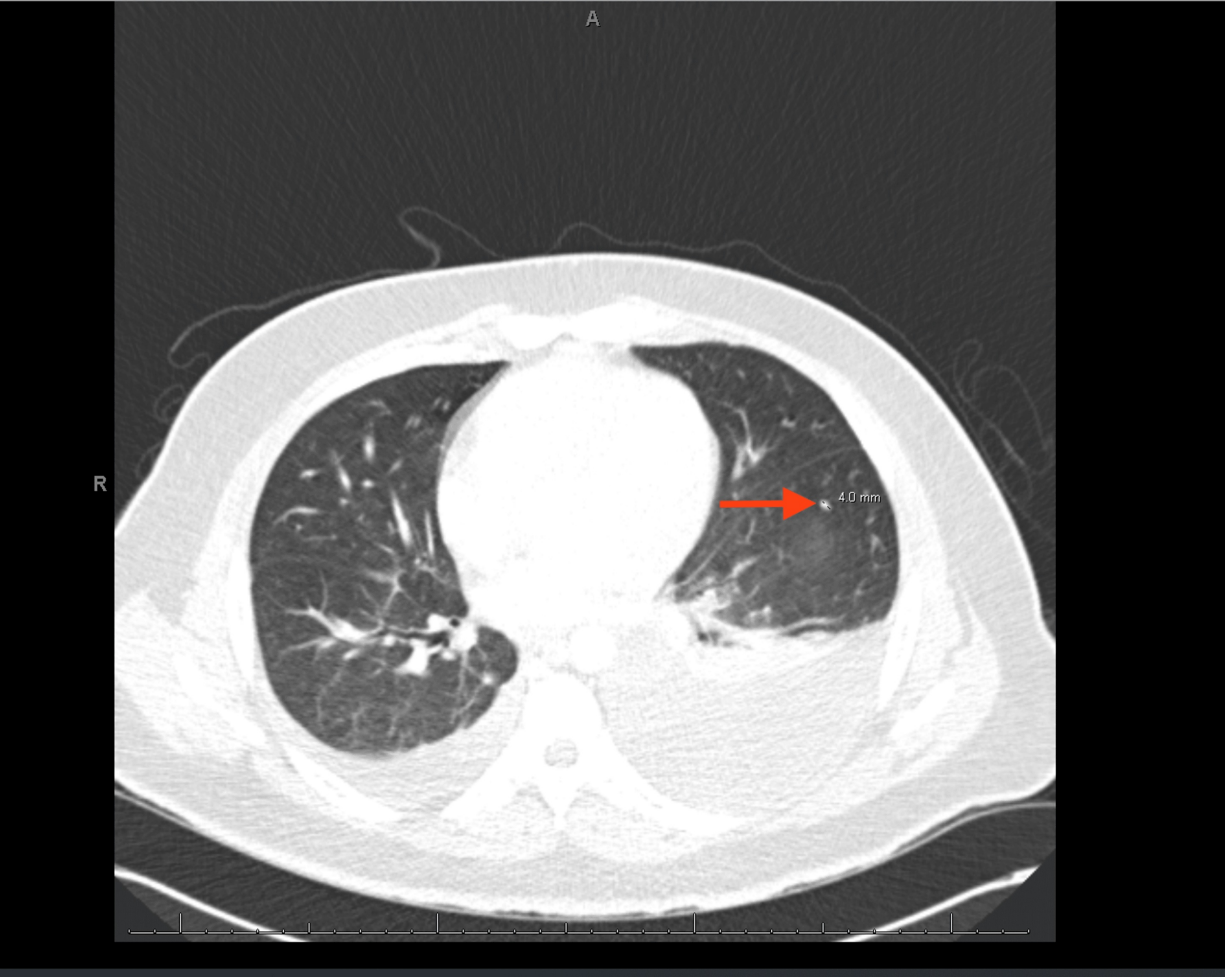
CT scan showing a 4 mm pulmonary nodule. The red arrow indicates a pulmonary nodule on the left lung.

**Figure 4:**
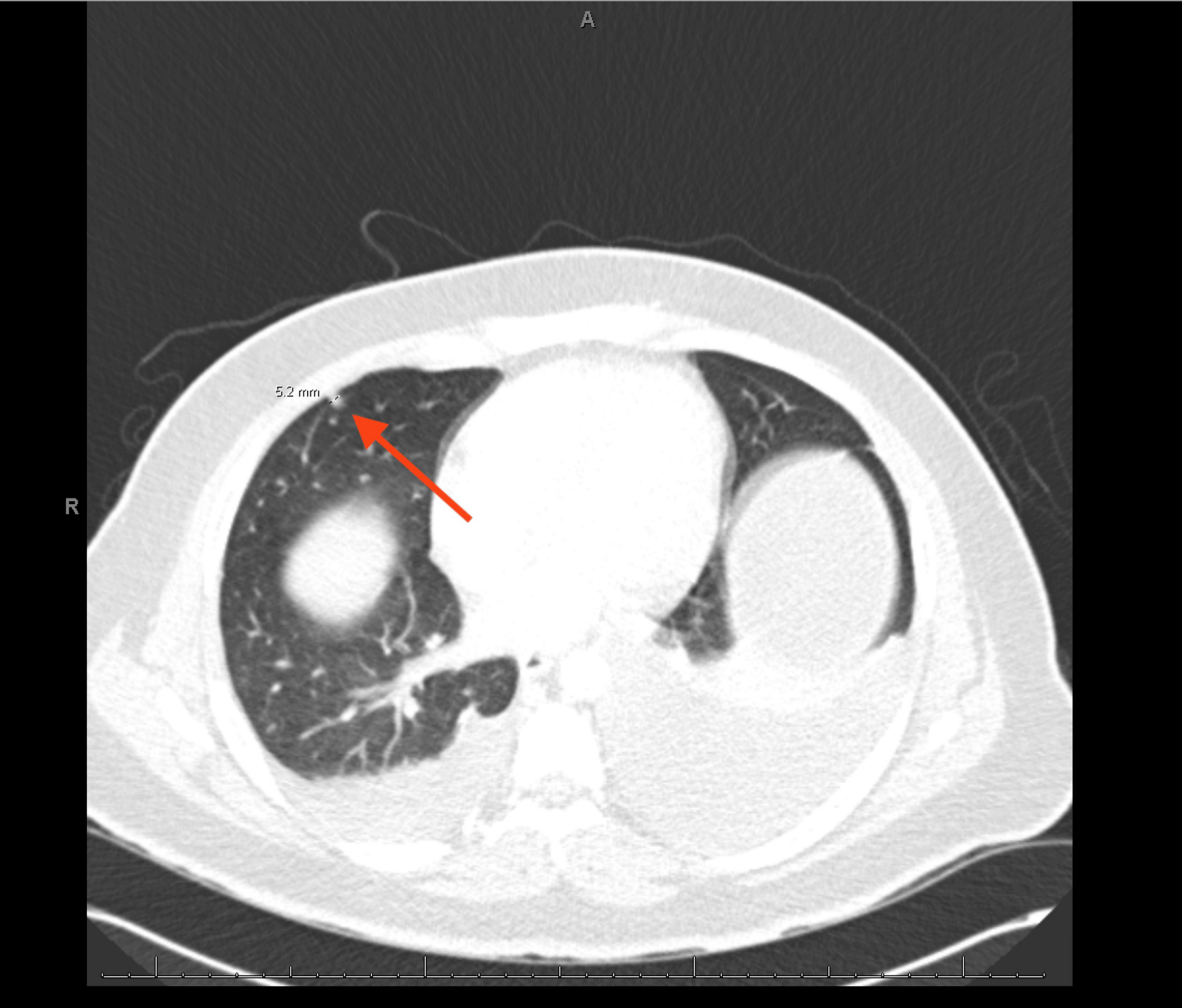
CT scan showing a 5 mm pulmonary nodule. The red arrow indicates a 5 mm pulmonary nodule in the right lung.

**Figure 5:**
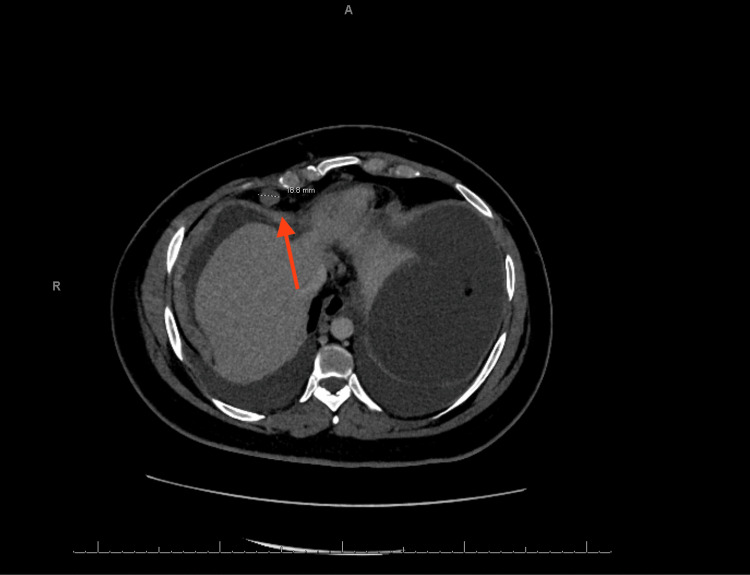
CT scan showing right cardiophrenic lymphadenopathy. The red arrow indicates a 19 mm lymphadenopathy.

**Figure 6:**
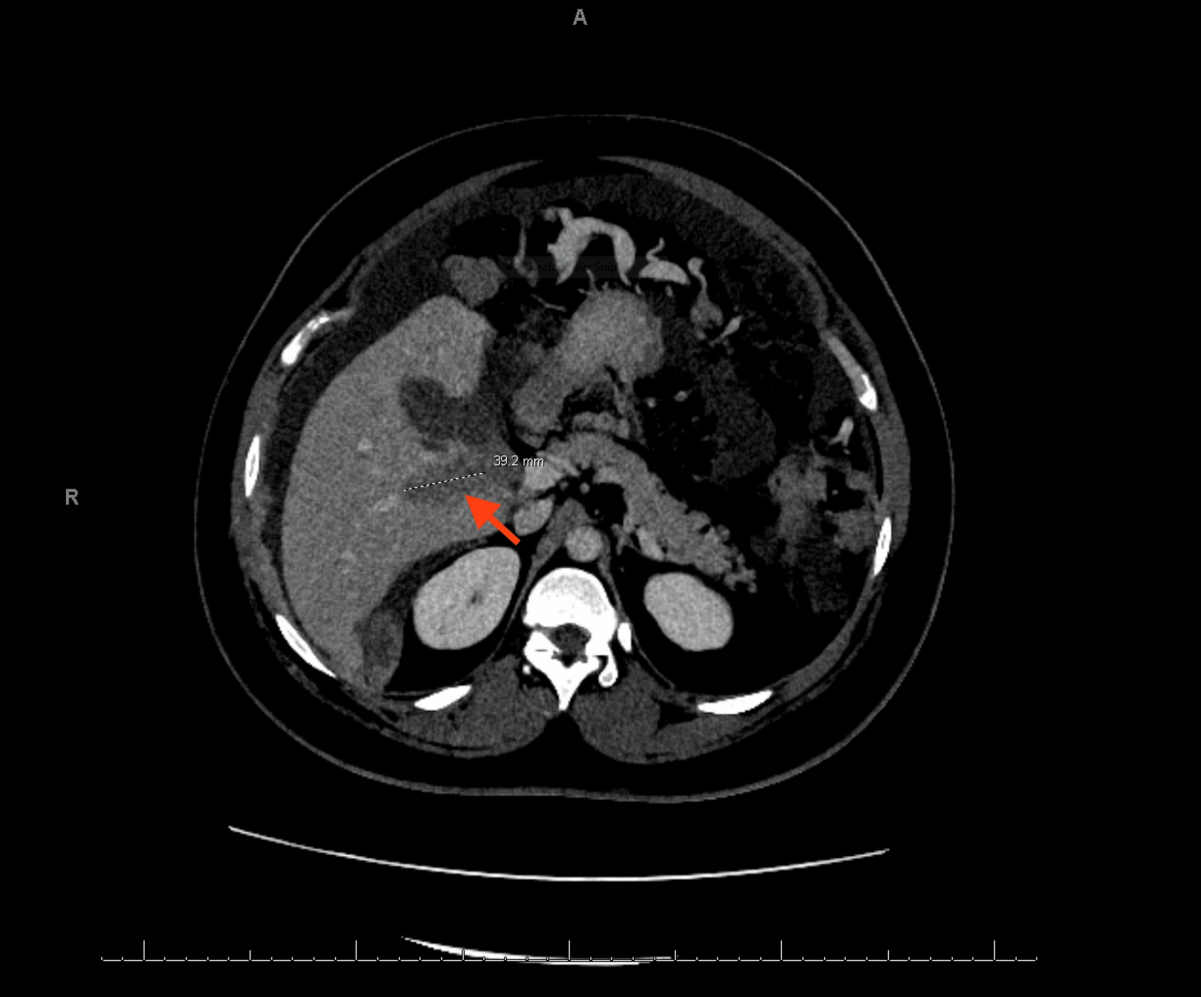
CT scan showing a 40 mm liver lesion. The red arrow indicates the metastatic spread of the tumor to the liver.

A CT of the chest with contrast revealed bilateral moderate to large volume pleural effusion, multiple pulmonary nodules up to 5 mm, suggestive of metastatic disease (Figures 3, 4), right cardiophrenic lymphadenopathy (Figure 5), liver lesions (Figure 6), and diffuse nodular thickening of the right hemidiaphragm.

A lymph node retroperitoneal biopsy was performed, revealing a malignant small round cell neoplasm consistent with a DSRCT. Immunohistochemistry showed specific positivity for CD56, focal positivity for pankeratin and synaptophysin, and dot-like positivity for desmin. The tumor cells were negative for chromogranin, CDX2, CK7, CK20, PAX8, TTF1, INSM1, and WT c-terminus. The Ki67 proliferation rate was greater than 90%.

Treatment was initiated with a chemotherapy regimen consisting of vincristine, doxorubicin, and cyclophosphamide (VDC) alternating with ifosfamide and etoposide (IE). A total of 10 cycles were planned. The patient received his first cycle and was monitored for three days, during which time he experienced nausea but noted a decrease in abdominal pain.

Upon discharge, the patient was prescribed pegfilgrastim to reduce the risk of febrile neutropenia and antiemetic medication to manage chemotherapy-induced nausea and vomiting. The importance of adhering to the treatment schedule was emphasized.

As of the current report, the patient has completed four cycles of the planned 10-cycle regimen. Regular follow-up appointments are being maintained to monitor treatment response and manage side effects. Preliminary observations indicate that while the patient has been experiencing nausea and difficulty eating, he reports less abdominal and back pain.

The long-term prognosis and outcomes for this patient remain to be determined as treatment is ongoing. Future directions include completion of the full chemotherapy regimen, regular imaging studies to assess tumor response, evaluation for potential surgical intervention, and monitoring of long-term survival and quality of life outcomes. This case highlights the importance of continued follow-up and comprehensive care in managing DSRCT.

## Discussion

Pathophysiological mechanisms

DSRCT is a rare and aggressive soft-tissue sarcoma characterized by the EWSR1-WT1 gene fusion. This unique genetic alteration leads to the upregulation of several growth factors and signaling pathways, contributing to the tumor’s aggressive behavior [4]. The EWSR1-WT1 fusion protein acts as a transcriptional activator, inducing the expression of genes involved in cell proliferation, angiogenesis, and invasion, such as *PDGFRA*, *IGF1R*, and *VEGF* [11].

The complex pathophysiology of DSRCT involves multiple interconnected mechanisms. The EWSR1-WT1 fusion protein activates various oncogenic pathways, including PI3K/AKT/mTOR and MAPK cascades, promoting cell survival and proliferation [11]. The characteristic desmoplastic stroma, rich in extracellular matrix proteins, creates a supportive microenvironment for tumor growth and may contribute to drug resistance. Recent studies suggest that DSRCT cells exhibit features of epithelial-mesenchymal transition which is linked to increased invasiveness, potentially explaining their aggressive metastatic behavior [4].

Diagnostics

This case highlights the diagnostic challenges of DSRCT, exemplified by the patient’s nonspecific abdominal symptoms persisting for over a year, reflecting the typical insidious onset often leading to delayed diagnosis [1]. DSRCT diagnosis combines clinical presentation, imaging, and pathological analysis.

CT and MRI are crucial for disease staging and characterization, revealing extensive peritoneal involvement with a dominant pelvic mass, multi-station lymphadenopathy, and pulmonary nodules indicating metastatic spread. MRI showed heterogeneous enhancement with central necrosis, typical of aggressive malignancies [4]. While suggestive, these imaging features are not pathognomonic.

Definitive diagnosis relied on histopathology, revealing the classic appearance of malignant small round cells, a feature shared by several malignancies [6]. The immunohistochemical profile was key in narrowing the differential diagnosis. Positivity for CD56, focal positivity for pan-keratin and synaptophysin, and the characteristic dot-like positivity for desmin are highly suggestive of DSRCT [10]. Notably, the exceptionally high Ki67 proliferation rate (>90%) observed in our patient’s tumor indicates its aggressive biological behavior and may correlate with poor prognosis [5]. This finding emphasizes the need for prompt and intensive therapeutic intervention.

While our case did not utilize fluorescence in situ hybridization (FISH), this technique could be valuable in future cases for detecting the EWSR1-WT1 gene fusion. FISH is highly sensitive and specific for identifying this pathognomonic genetic alteration in DSRCT. Implementing FISH in the diagnostic workflow could provide a more rapid and definitive diagnosis, potentially expediting treatment initiation and opening avenues for targeted therapies and clinical trial eligibility [11].

The diagnostic journey in this case demonstrates the importance of a stepwise, multimodal approach to diagnosing DSRCT, emphasizing the need for high clinical suspicion, especially in young male patients with peritoneal masses. Moving forward, regular imaging surveillance will be crucial in monitoring treatment response and detecting disease progression, guiding timely adjustments to the therapeutic strategy

Treatment plans

Management of DSRCT requires a comprehensive, multidisciplinary approach. The current standard of care involves intensive multiagent chemotherapy (e.g., VDC/IE regimen), aggressive surgical resection, and radiation therapy [10]. Emerging targeted therapies include tyrosine kinase inhibitors such as pazopanib, which target vascular endothelial growth factor and platelet-derived growth factor receptors and have shown promise in clinical studies. mTOR inhibitors such as temsirolimus have demonstrated some efficacy, especially in combination with other targeted therapies. Antibodies targeting insulin-like growth factor-1 receptor, such as ganitumab, have shown clinical benefit in some patients [11].

Immunotherapy is also being explored in DSRCT treatment. Checkpoint inhibitors such as nivolumab and ipilimumab have shown potential in early studies, albeit in select cases [11]. Novel approaches under investigation include strategies targeting the EWSR1-WT1 fusion protein, epigenetic modifiers, and neurotrophic tropomyosin receptor kinase (NTRK) inhibitors.

Disease progression and outcomes

Despite aggressive multimodal therapy, the prognosis for DSRCT remains poor. Most patients experience disease progression or recurrence within the first three years after initial treatment [11]. The aggressive nature of DSRCT, its propensity for early metastasis, and the development of treatment resistance contribute to these poor outcomes.

Factors influencing prognosis include the extent of disease at diagnosis, completeness of surgical resection, and response to initial chemotherapy. Patients with localized disease and those achieving complete surgical resection tend to have better outcomes, although long-term survival remains rare [10].

Future directions

The future of DSRCT treatment lies in unraveling its complex biology and developing targeted, personalized approaches. Key areas of research include targeting the EWSR1-WT1 fusion protein or its downstream effectors, with promising advances in protein degradation technologies such as PROTACs [4]. Researchers are also exploring epigenetic modifiers to counteract the effects of the EWSR1-WT1 fusion protein [5].

Liquid biopsy techniques are also showing promise. Campos et al. (2020) demonstrated the potential of tracking the EWSR1-WT1 fusion in circulating tumor DNA as a biomarker for minimal residual disease. This approach could allow for more personalized treatment decisions and earlier detection of relapse.

Liquid biopsy techniques show potential for tracking the EWSR1-WT1 fusion in circulating tumor DNA, enabling personalized treatment decisions and early relapse detection [12]. Following the discovery of EWS-WT1's role in NTRK3 transcription, NTRK inhibitors are being investigated as potential therapies [4]. Additionally, large-scale genomic and functional studies, such as CRISPR-Cas9 screens, aim to identify novel therapeutic targets. These efforts may pave the way for more effective, personalized treatment strategies.

In conclusion, while the current management of DSRCT remains challenging, ongoing research into its unique biology and the development of targeted therapies offer hope for improving outcomes. Future efforts should focus on translating these biological insights into DSRCT-specific clinical trials, improving early detection methods, and developing more effective, personalized treatment strategies for this aggressive malignancy.

## Conclusions

This case highlights the challenges in diagnosing and managing DSRCT, emphasizing the importance of maintaining a high index of suspicion in young adults with vague abdominal complaints that fail to resolve. The extensive disease found at diagnosis highlights the need for early detection and diagnostic tools. Current imaging and biopsy guidelines, while crucial for diagnosis, often result in late-stage detection due to the tumor’s nonspecific and subtle progression of symptoms.

Despite aggressive multimodal treatment, which consists of chemotherapy, surgery, and radiation, the prognosis for advanced DSRCT remains poor with low survival rates. This case emphasizes the limitations of the current therapeutic approaches. The poor outcomes seen in both this case and in the literature indicate the need to find effective treatment options.

Future research should focus on developing more sensitive and specific diagnostic tools to facilitate earlier detection via genetic testing or biomarkers, such as the EWSR1-WT1 gene fusions that are found to be detectable by using liquid biopsy techniques. Additionally, immunotherapy has shown promise as a potential future treatment option. Research is being conducted on the exploration of novel targeted therapies that aim at the pathways that are activated due to this fusion. Further investigation needs to also be conducted on optimizing the timing and sequencing of multimodal treatments, as well as identifying biomarkers to monitor treatment response. These future investigations could potentially improve outcomes and quality of life for patients diagnosed with this rare and aggressive malignancy.
